# Extremely High Mutation Rate of HIV-1 In Vivo

**DOI:** 10.1371/journal.pbio.1002251

**Published:** 2015-09-16

**Authors:** José M. Cuevas, Ron Geller, Raquel Garijo, José López-Aldeguer, Rafael Sanjuán

**Affiliations:** 1 Instituto Cavanilles de Biodiversidad y Biología Evolutiva, Valencia, Spain; 2 Hospital Universitario La Fe, Valencia, Spain; 3 CoRIS and HIV Biobank, Spanish AIDS Research Network, Spain; 4 Departament de Genètica, Universitat de València, Valencia, Spain; Weatherall Institute of Molecular Medicine, UNITED KINGDOM

## Abstract

Rates of spontaneous mutation critically determine the genetic diversity and evolution of RNA viruses. Although these rates have been characterized in vitro and in cell culture models, they have seldom been determined in vivo for human viruses. Here, we use the intrapatient frequency of premature stop codons to quantify the HIV-1 genome-wide rate of spontaneous mutation in DNA sequences from peripheral blood mononuclear cells. This reveals an extremely high mutation rate of (4.1 ± 1.7) × 10^−3^ per base per cell, the highest reported for any biological entity. Sequencing of plasma-derived sequences yielded a mutation frequency 44 times lower, indicating that a large fraction of viral genomes are lethally mutated and fail to reach plasma. We show that the HIV-1 reverse transcriptase contributes only 2% of mutations, whereas 98% result from editing by host cytidine deaminases of the A3 family. Hypermutated viral sequences are less abundant in patients showing rapid disease progression compared to normal progressors, highlighting the antiviral role of A3 proteins. However, the amount of A3-mediated editing varies broadly, and we find that low-edited sequences are more abundant among rapid progressors, suggesting that suboptimal A3 activity might enhance HIV-1 genetic diversity and pathogenesis.

## Abstract 2

The rate of spontaneous mutation of the HIV-1 genome within its human host is exceptionally high, is mostly driven by host cytidine deaminases, and probably plays a role in disease progression.

## Author Summary

The high levels of genetic diversity of the HIV-1 virus grant it the ability to escape the immune system, to rapidly evolve drug resistance, and to circumvent vaccination strategies. However, our knowledge of HIV-1 mutation rates has been largely restricted to in vitro and cell culture studies because of the inherent complexity of measuring these rates in vivo. Here, by analyzing the frequency of premature stop codons, we show that the HIV-1 mutation rate in vivo is two orders of magnitude higher than that predicted by in vitro studies, making it the highest reported mutation rate for any biological system. A large component of this rate is from host cellular cytidine deaminases, which induce mutations in the viral DNA as a defense mechanism. While the HIV-1 genome is hypermutated in blood cells, only a very small fraction of these mutations reach the plasma, indicating that many viruses are defective as a result of the extremely high mutation load. In addition, we find that the HIV-1 mutation rate tends to be higher in patients showing normal disease progression than in those undergoing rapid progression, emphasizing the negative impact on viral fitness of hypermutation by host cytidine deaminases. However, we also observe subpopulations of weakly-mutated viral genomes whose sequence diversity may influence viral pathogenesis. Our work highlights the fine balance for HIV-1 between enough mutation to evade host responses and too much mutation that can inactivate the virus.

## Introduction

RNA viruses exist as extremely diverse populations, with every possible spontaneous mutation along the genome appearing within each patient every day [1]. This diversity plays a fundamental role in HIV-1 biology, enabling the virus to successfully evade the immune system, rapidly modify cell tropism, evolve drug resistances, and thwart vaccination strategies [2,3]. Similar to other RNA viruses, the great diversity of HIV-1 stems from its high rate of spontaneous mutation, defined as the probability that a new mutation appears per base in each infected cell [4,5]. The HIV-1 reverse transcriptase (RT) lacks proofreading activity and has an estimated error rate on the order of 3 × 10^−5^ per base per round of copying as determined in cell culture studies [4,6–9]. However, these estimates may not truly reflect the mutational process of HIV-1 in patients, because cellular factors such as dNTP levels or sequence context can affect the frequency and type of mutations produced [10–12]. Furthermore, HIV-1 is subject in vivo to editing by cellular enzymes of the apolipoprotein B mRNA editing enzyme, catalytic polypeptide-like 3 (A3) family, which are packaged into the virion and, upon infection of a new cell, mediate the edition of cytidine to uracil in the negative-strand viral cDNA, resulting in G→A substitutions in the viral genomic RNA [13–15]. Numerous A3 enzymes are expressed in humans, but A3D, A3F, A3G and A3H are believed to be the most relevant to HIV-1 pathogenesis [14,15]. Their antiviral role is underscored by the function of the HIV-1 Viral infectivity factor (Vif), which promotes A3 proteasomal degradation and is required for infectivity in CD4 cells [13]. Multiple studies have demonstrated the existence of high levels of A3-induced substitutions in patient-derived HIV-1 sequences (hypermutation), but the contribution of A3 to the total mutation rate of the virus has not been quantified. Also, conflicting results have been reported regarding the role played by A3 in HIV-1 diversity and evolution. While cell culture experiments have suggested that hypermutation is invariantly lethal for the virus [16], others have reported that A3 can promote immune escape and drug resistance [17,18]. Interestingly, A3 expression differs among patients, and recent work has shown that at least seven A3H haplotypes with different geographic distributions exist, of which only three represent stable enzymes capable of editing HIV-1 but can be counteracted by some Vif variants [14,19–22]. However, the role played by A3 edition in disease progression still remains debated, with some studies suggesting a positive association between A3 activity and clinical outcome [23–25], but not others [26]. Hence, the HIV-1 mutation rate in vivo, the contribution of the HIV-1 RT, and host A3 proteins to this rate, as well as its relevance to disease progression, remain to be elucidated.

In this study, we estimate the mutation rate of HIV-1 using sequences derived from both intracellular viral DNA and plasma viral RNA. Although many factors determine the genetic diversity of a virus, including, among others, natural selection, transmission bottlenecks, and cell turnover rates, the effect of mutation rate on genetic diversity can be disentangled from these other factors by focusing on mutations that abrogate viral infectivity (lethal mutations) [27]. We thus performed massive parallel sequencing of the entire HIV-1 protein coding region from 11 infected donors, obtained a high confidence set of 3,069 likely lethal mutations in viral DNA from cells, and contrasted these results with those obtained from previously published HIV-1 sequences derived from plasma RNA. Whereas the observed mutation rate in plasma was compatible with the known fidelity of the HIV-1 RT, our results reveal a strong inflation of the mutation rate in DNA sequences, with an estimated (4.1 ± 1.7) × 10^−3^ mutations per base per cell. By examining the relative contribution of the HIV-1 RT, A3G, and A3D/F/H to the overall mutation rate of the virus, we show that this extremely high rate is essentially driven by the action of A3 enzymes. Supporting the antiviral role of A3-mediated HIV-1 genome editing, we find a significantly lower viral mutation rate in patients showing rapid disease progression compared to normal progressors and a negative correlation between the viral mutation rate and the set-point viral load. However, the extent of A3 editing varies broadly among sequences, with some sequences showing only few A3-driven mutations. Interestingly, we find that low-level A3 editing is more abundant in rapid progressors than in normal progressors, suggesting that failure of A3 to inactivate the virus by hypermutation may promote HIV-1 intrapatient diversity and pathogenesis.

## Results

### Inference of the HIV-1 Mutation Rate In Vivo

To infer the HIV-1 mutation rate from patient-derived sequences, we used the lethal mutation method [27], which builds on the principle that the frequency of lethal mutations in a population equals their rate of production, as these cannot be inherited. We used premature stop codons as a surrogate for lethal mutations, and the mutation rate was calculated by dividing the observed number of stops by the total number of possible single-base substitutions leading to stop codons (nonsense mutational targets, NSMTs; see methods). NSMTs are distributed throughout protein-coding regions, thus allowing for a genome-wide assessment of the mutation rate. When we applied the lethal mutation method to three large available datasets of HIV-1 sequences from plasma RNA (2689 subtype B *env*, 1450 subtype C *env*, and 1310 *gag* sequences from either subtype), we obtained a mutation rate of (9.3 ± 2.3) × 10^−5^ per base per cell, which is slightly higher than those reported in cell culture studies [4]. However, A3-driven mutations may be largely absent from plasma because selection against highly mutated sequences may impede viral assembly and budding [28,29]. We therefore reasoned that focusing on plasma RNA may underestimate the mutation rate in vivo. Hence, we amplified the entire protein-coding region of the viral DNA directly from peripheral blood mononuclear cells (PBMCs) of 11 treatment-naive patients with known infection, viral load, and CD4 count histories (Table 1, S1 Fig, S2 Fig). High-fidelity limiting-dilution PCR was carried out in three overlapping fragments. Since in this method each positive PCR is effectively initiated from a single template molecule, cloning takes place before PCR, thus preventing the accumulation of PCR errors and amplification biases [30–34]. Using DNA as template, rather than RNA, has the additional advantage of avoiding the RT step prior to PCR, which is a major source of errors resulting from the low in vitro fidelity of these enzymes [35,36]. For each patient, 50 limiting-dilution clonal PCR products were obtained per fragment, thereby enabling us to analyze 11 × 50 = 550 full-length protein-coding sequences.

**Table 1:** Patient clinical data and mutation rate summary.

Patient	Sex	Age	Infection time (years)	CD4count (cell/μL)	Per-year CD4 count decay rate (cell/μL)^a^	log_10_ set-point viral load (copies/mL)^b^	Progression rate^c^	HIV-1 mutation rate per base per cell
**R3**	M	22	1.31	380	321 ± 82	5.4 ± 0.2	Rapid	1.9 × 10^−3^
**R5**	M	37	1.82	432	183 ± 41	5.1 ± 0.1	Rapid	1.6 × 10^−3^
**R6**	M	42	3.10	218	243 ± 39	4.6 ± 0.3	Rapid	3.7 × 10^−3^
**R7**	M	54	1.63	291	459 ± 33	5.1 ± 0.1	Rapid	2.8 × 10^−3^
**R8**	M	25	1.00	338	160	4.4	Rapid	2.4 × 10^−3^
**R11**	M	52	0.96	689	87 ± 25	4.0 ± 0.2	Normal	3.4 × 10^−3^
**R14**	M	31	NA	637	76 ± 22	4.1 ± 0.2	Normal	3.7 × 10^−3^
**R15**	M	25	1.24	446	68 ± 25	4.2 ± 0.1	Normal	12.6 × 10^−3^
**R4**	M	26	2.16	439	48 ± 57	4.3 ± 0.1	Normal	3.2 × 10^−3^
**R9**	M	35	0.65	1146	124 ± 111	3.9 ± 0.1	Normal	4.8 × 10^−3^
**P6**	F	49	20.94	431	37 ± 4	4.3± 0.1	Normal	4.5 × 10^−3^

^a^ Estimated by linear regression as the slope of CD4 counts against infection time. For R8, although there were few data points for reliably estimating the CD4 decay rate, rapid progression was supported by the observation that the CD4 count dropped to 338 cells/μL within the first year of infection. Full data are shown in S1 Fig, the S1 Data file, and the S2 Data file.

^b^ Average log viral load obtained from all available patient samples taken at least one year postinfection but before the onset of treatment and/or symptoms. R8 had only one available viral load determination. For R9, a viral load determination taken 329 d after infection was included in the calculation to have at least three data points. Full data are shown in S2 Fig, the S1 Data file, and the S2 Data file.

^c^ Rapid progressors were defined as patients showing a CD4 count decay rate greater than 150 cell/μL per y.

**Fig 1:**
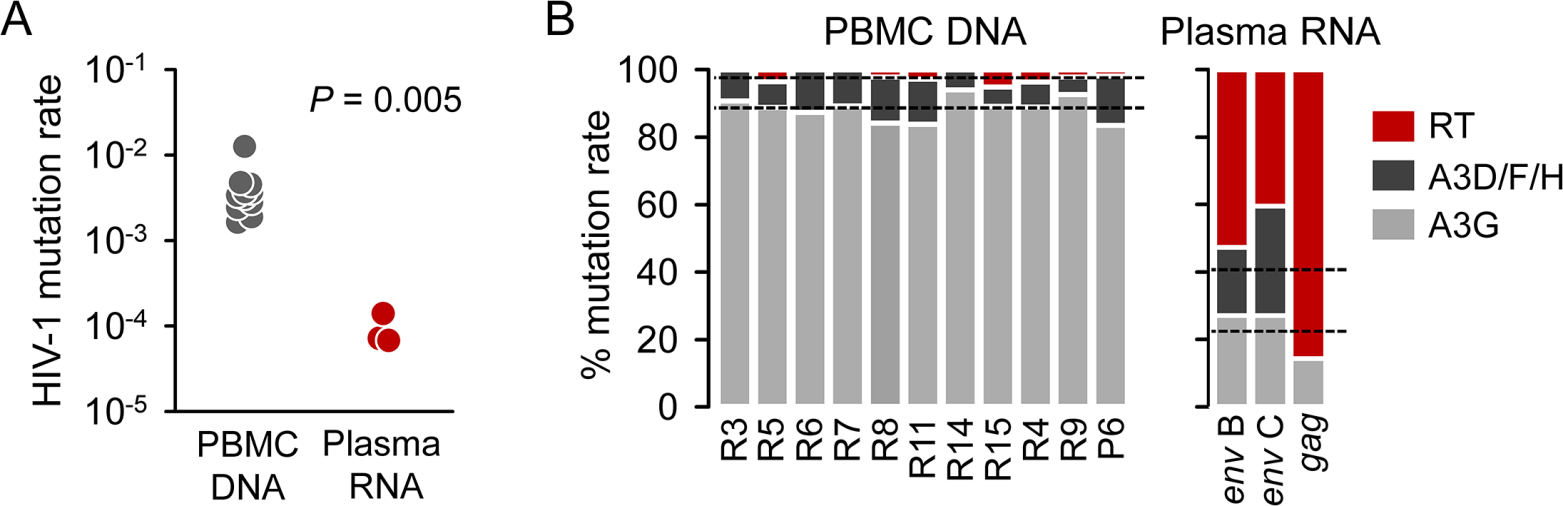
HIV-1 mutation rate and source of mutations in DNA from PBMCs and in RNA from plasma. **A.** The mutation rate was inferred from PBMC DNA and from plasma RNA using the lethal mutation method. Each data point represents the average mutation rate obtained for one patient (PBMC DNA) or the average rate calculated from one large publicly available dataset of *gag* or *env* sequences (plasma RNA). **B.** Contribution of A3G, A3D/F/H, and the viral RT to the observed mutation rate in PBMC DNA and plasma RNA sequences. Dotted lines indicate the average contribution of each enzyme. Individual numerical values shown in this Figure are available in Table 1 and in the S1 Data file.

For practical reasons, PCRs were arranged in 110 libraries each containing an equimolar pool of five PCRs per fragment representing five full-length protein-coding regions, and libraries were uniquely tagged and subjected to Illumina paired-end sequencing. As a control, we similarly amplified 50 HIV-1 protein-coding regions from the reference plasmid pNL4-3. An average coverage of 19,150 ± 5,801 reads per site was achieved (minimum of 4,000), and mutations relative to the patient consensus sequence were then called. Application of the lethal mutation method to the control pNL4-derived sequences yielded no mutations, as expected. In contrast, in samples from the 11 patients, we found 3,069 total stop-codon mutations in the 732,350 NSMTs examined, giving a per-patient average mutation rate of (4.1 ± 1.7) × 10^−3^ per base per cell or, equivalently, one mutation every 250 bases. This value is 44 times greater than the rate observed in plasma samples (Fig 1A) and approximately two orders of magnitude higher than the rates reported previously in cell culture studies.

### Contribution of A3 and RT to the HIV-1 Mutation Rate

The sequence contexts for cDNA editing by different A3 enzymes have been previously defined in detail [13,14,37,38]. A3G shows a strong preference for CC sequences on minus-strand cDNA leading to GG → AG mutations in the positive strand. In contrast, GA → AA editing is largely mediated by A3D/F/H. Hence, A3 activity leads to stop codons by edition of tryptophan TGG codons, with A3G and A3D/F/H leading to different stop codons depending on sequence context. A3G edition mutates TGG codons to TAG stops, and to either TGA or TAA stops if the TGG codon is followed by a G (TGGG), whereas A3D/F/H lead to TGA stops if the TGG codon is followed by an A (TGGA). In contrast, the HIV-1 RT can mediate all possible mutations at both TGG codons as well as numerous other codons. Using these sequence context preferences allowed us to assign mutations to A3G, A3D/F/H, or the HIV-1 RT (**Table 2**).

**Table 2:** Assignment of mutations in NSMTs to A3G, A3D/F/H, or RT.

NSMT	Resulting stop codon^a^		
	TAG	TGA	TAA
TGG A	A3G	A3D/F/H	A3G + A3D/F/H
TGG C	A3G	RT	A3G + RT
TGG G	A3G	A3G	A3G + A3G
TGG T	A3G	RT	A3G + RT
Other (17 codons)	RT	RT	RT

^a^In all cases the indicated mutations can also be produced by the RT in addition to A3 enzymes.

Analysis of sequences from viral RNA suggested a relatively balanced contribution of A3G, A3D/F/H, and HIV-1 RT to the total mutation rate (22.8 ± 4.2%, 17.6 ± 9.5%, and 59.7 ± 13.5%, respectively, assuming that all G→A changes falling at canonical A3 targets were produced by A3 and not by the HIV-1 RT; Fig 1B). However, these percentages may be inaccurate due to the low fidelity of the RT-PCR step needed for sequencing of viral RNA [35]. In contrast, in viral DNA sequences from PBMCs, A3G contributed 88.4 ± 1.1% of the total mutation rate, A3D/F/H contributed 9.7 ± 1.1%, and the HIV-1 RT only 2.0 ± 0.54% (Fig 1B). Among the total 56,800 NSMTs available for A3G editing in the viral DNA sequences from the 11 patients, 2,752 contained stop codon mutations, the fraction of A3G targets edited being thus 4.8%. This shows that the extremely high mutation rate observed in viral DNA was essentially driven by A3 edition, and that sequences edited by A3 were comparatively much more difficult to sample in RNA sequences from plasma than nonmutated or RT-mutated sequences. Indeed, the observed RT mutation rate was nearly identical in DNA (6.3 × 10^−5^) and plasma RNA sequences (6.2 × 10^−5^). In theory, G→A changes falling at canonical A3 targets could also be produced by the RT. However, since mutations were clearly concentrated in these targets and the RT does not show such sequence-context preferences [36], the vast majority of mutations should be correctly assigned to A3. Further confirming our assignment of mutations, the observed edits were consistent with known A3 sequence-context preferences beyond the GG and GA dinucleotides [37,38]. Specifically, A3G mutations were enhanced by the presence of a T at position –2 relative to the mutated G (TNGG; Binomial test: *p* < 0.01 in 6 of 11 patients), a T at position –1 (TGG; *p* < 0.01 in 11 of 11 patients), and a G at position +2 (GGG; *p* < 0.01 in 10 of 11 patients), whereas they were negatively influenced by an A or a G at position –1 (A/GGG; *p* < 0.01 in 7 and 9 of 11 patients, respectively) and a C at position +2 (GGC; *p* < 0.01 in 10 of 11 patients). In turn, A3D/F/H mutations were also favored by a T at position –1 (TGA; *p* < 0.01 in 7 of 11 patients) and negatively affected by a C at position –2 (CNGA; *p* < 0.01 in 7 of 11 patients) or a C at position +2 (GAC; *P* < 0.01 in 9 of 11 patients).

### Variation in the HIV-1 Mutation Rate

**Fig 2:**
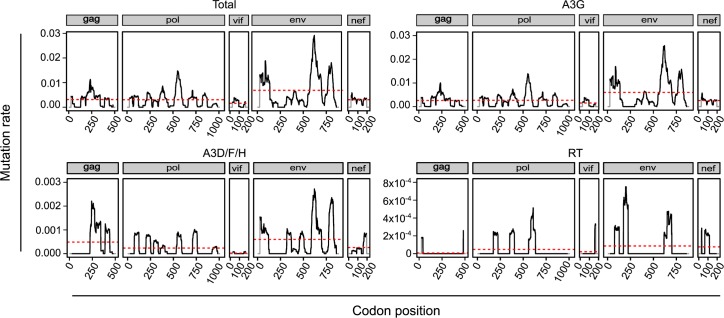
Distribution of mutation rates across HIV-1 genes. The average total, A3G, A3D/F/H, and RT mutation rate within a sliding window of 50 codons across HIV1 genes (black skyline), and the average mutation rate for each gene (red dashed line) are shown. Stop codon and NSMT counts are represented in S3 Fig, and numerical values are available from the S3 Data file.

In our dataset, stop codons were observed throughout the viral genome, and their abundance tended to vary accordingly with the number of available NSMTs, as expected (S3 Fig). Calculation of the mutation rate (stops/NSMTs) using a 50-codon sliding window revealed several regions with a higher-than-average rate, which mapped mainly to gp41, the N-terminal region of gp120, and the central regions of *gag* and *pol* (Fig 2). Comparison of mutation rates for the major five genes (*gag*, *pol*, *vif*, *env*, and *nef*) also yielded significant differences (Kruskal-Wallis test: *p* < 0.001), the rate being highest in gp41 and lowest in *vif* (Tukey HSD posthoc test: *p* < 0.05). A3 editing depends on the amount of time the viral DNA is found as single-stranded DNA, the preferred A3 substrate, and this produces twin-peaks gradients of A3 edition, which are largely consistent with our observed distribution of mutation rates [37,38].

**Fig 3:**
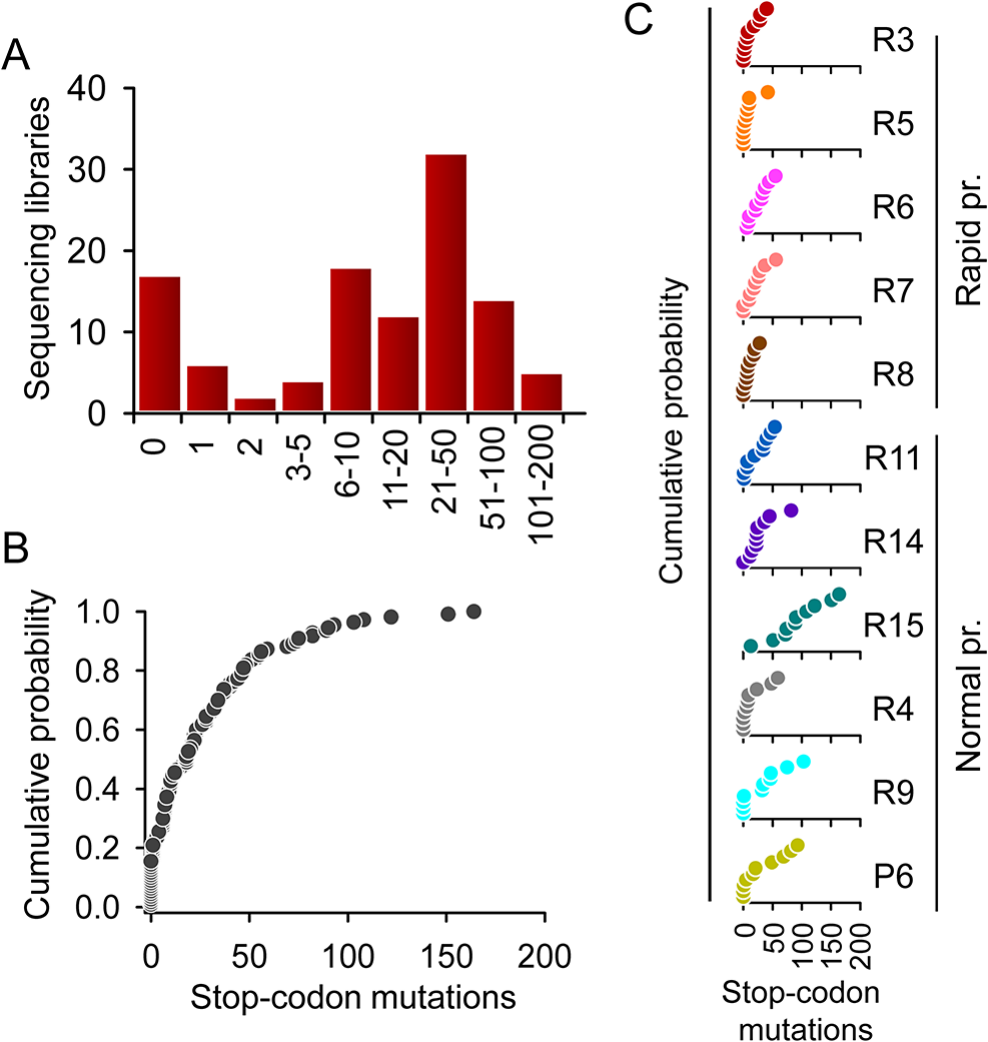
Variation in mutation rate among HIV-1 sequences. **A.** Histogram showing the distribution of the number of stop codon mutations per sequencing library. **B and C.** Cumulative probability distribution of the number of stop codon mutations per sequencing library for the entire dataset (B) and for each patient (C). Notice that the number of mutations tends to be higher in normal progressors (bottom) than in rapid progressors (top). Individual numerical values shown in this Figure are available in the S4 Data file.

In addition to the across-genome mutation rate variation, we were interested in characterizing how the number of mutations varied among sequencing libraries. The effects of A3 edition on viral population fitness may be relatively minor if the vast majority of mutations were clustered in a small subset of sequences, whereas they should be substantial otherwise. While an all-or-nothing model has been suggested wherein any HIV-1 genome subjected to editing is so extensively mutated that it has an exceedingly low probability of being viable [16], other works have shown varying levels of A3 editing [17,18]. To address this, we first examined the number of total, A3G-, and A3D/F/H-driven lethal mutations in each of the 110 sequencing libraries. We found that only 17 libraries (15.4%) were free of stop codon mutations and that, among the rest of the libraries, mutation counts varied gradually across two orders of magnitude (from 1 to 164), arguing against an all-or-nothing pattern (Fig 3A and 3B). Examination of each patient separately also indicated a large variation in the number of mutations and that mutation-free libraries were rare (0% to 30% depending on the patient; Fig 3C).

However, since each library was made of five limiting-dilution PCRs, these data did not have enough resolution to assess the number of mutations per individual sequence. To address this, we performed a more detailed, intralibrary analysis of mutations for the *nef* gene. In total, there were 147 *nef* premature stop codon mutations in 45,400 NSMTs, giving a mutation rate of 3.2 × 10^−3^ per base per cell. Of the 110 libraries, 31 contained two or more stop codon mutations in this single gene. For each of these 31 libraries, we sequenced the five limiting-dilution PCR clones of the library by the Sanger method to ascertain whether the multiple stops occurred in one or several clones. We found that in 13 libraries (42%), stop codon mutations were significantly clustered in only one or two of the clones (test against Poisson expectation: *p* < 0.05), whereas in the remaining 18 libraries (58%), we could not reject the possibility that mutations were randomly spread across clones (S4 Fig; S5 Data). These data confirm that there are hypermutated sequences but that A3 edition does not follow an all-or-nothing pattern in vivo.

### Correlation between the HIV-1 Mutation Rate and Disease Progression

**Fig 4:**
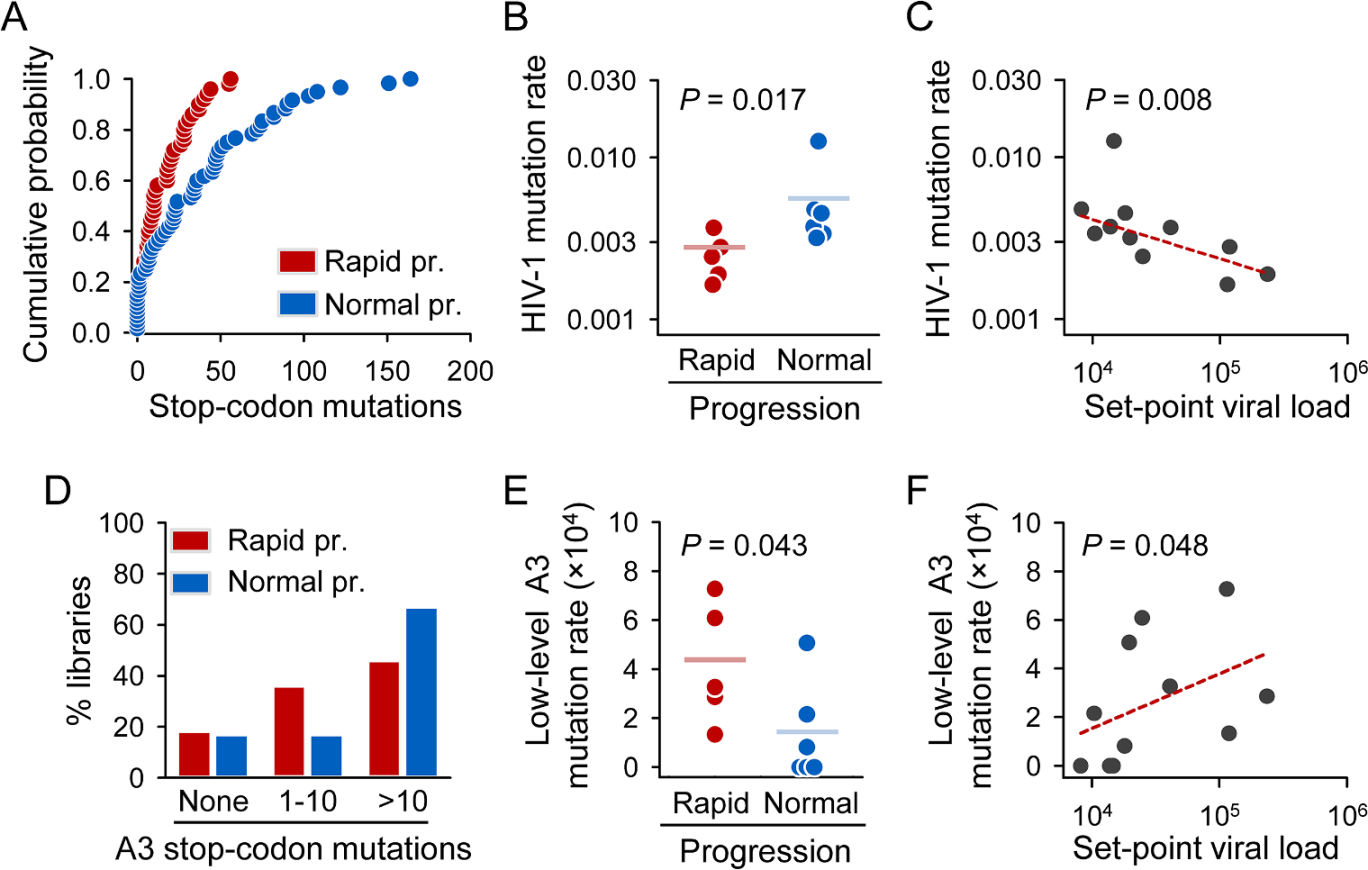
Association between HIV-1 mutation rate and disease progression markers. **A.** Cumulative distribution of the number of stop-codon mutations per sequencing library for rapid and normal progressors. **B.** Differences in mutation rate between these two patient groups. **C.** Inverse correlation between the HIV-1 mutation rate and the set-point viral load. The linear regression line obtained after removing the high-mutation outlier patient R15 is shown in red. **D.** Percent of libraries showing no (0), low-level (1–10), or high-level A3 editing (>10) in rapid and normal progressors. **E.** Differences in low-level A3 mutation rate (using libraries with 1–10 A3-driven mutations) between normal and rapid progressors. **F.** Positive correlation between the low-level A3 mutation rate and the set-point viral load. The linear regression line is shown in red. Individual numerical values shown in this Figure are available on Table 1 and in the S1 Data file.

Five of the patients were defined as rapid progressors based on CD4 count decay rates (>150 cells/μL per year), whereas the other six patients were normal progressors (Table 1). This grouping was supported by differences in set-point viral load, defined as the approximately stable viral load reached after acute infection but before the onset of symptoms and/or treatment. The viral load set-point has been shown to be a good predictor of disease progression [39,40]. Here, the set-point load averaged (1.1 ± 0.4) × 10^5^ copies/mL for rapid progressors, versus (1.4 ± 0.2) × 10^4^ copies/mL for normal progressors (Mann-Whitney test: *p* = 0.004). Despite the low number of patients in each group, we found several lines of evidence linking the HIV-1 mutation rate with disease progression. Firstly, the distribution of the number of stop-codon mutations per sequencing library differed between rapid and normal progressors (Fig 4A; Kolmogorov-Smirnov test: *p* = 0.003). Secondly, there was a statistically significant, 2.2-fold decrease in the HIV-1 mutation rate in rapid progressors compared with normal progressors, from (5.4 ± 3.6) × 10^−3^ to (2.5 ± 0.8) × 10^−3^ (Mann-Whitney test: *p* = 0.017; Fig 4B). Thirdly, we found that the mutation rate correlated negatively with the set-point viral load (Spearman *ρ* = –0.755; *p* = 0.007; Fig 4C). Examination of the distribution of mutations across the viral genome revealed that the difference between rapid and normal progressors was consistent in all genes except *gag*, in agreement with findings showing that A3G expression in infected individuals correlates with hypermutation levels in *vif* and *env* but not in *gag* [41] (S5 Fig).

Interestingly, the consensus viral sequence from patient R15 had a nonconservative amino acid replacement in the histidine(H)/cysteine(C)-containing (HCCH) motif of Vif (E134T), which has been previously implicated in the interaction of Vif with CBF-β, a requirement for A3 degradation [42]. As predicted from the anti-A3 effect of Vif, the HIV-1 mutation rate for this patient was four times higher than the average for the other patients (Table 1). Importantly, the association between disease progression and the HIV-1 mutation rate was not driven exclusively by this single data point, since significant differences were still observed after removal of this patient from the dataset (Mann-Whitney test: *p* = 0.030; correlation with set-point viral load: *ρ* = –0.782; *p* = 0.008). Finally, since A3H is the most genetically diverse of the seven human A3 genes, with some alleles encoding stable A3H forms while others encoding short-lived forms with little or no antiviral activity [22], we determined the A3H genotype of each patient. All patients were homozygous for an inactive A3H allele with the exception of R15. This patient was heterozygous for an active A3H allele (see Methods), but R15 Vif sequences contained the A3H susceptibility residue V39 [19–21], thus suggesting an additional mechanism for the extremely high HIV-1 mutation rate shown by R15. Although our results support the antiviral effect of A3-mediated hypermutation, previous work has suggested that low-level editing may also contribute to viral diversity and, potentially, to pathogenesis [17,18]. We found that the number of A3 mutations per library did not follow the same distribution in rapid and normal progressors. In rapid progressors, 36% of the libraries had between one and ten mutations (low-level editing), whereas this fraction dropped to 17% in normal progressors (Fisher test: *p* = 0.021; Fig 4D). Whereas highly-edited libraries (>10 stop-codon mutations) recapitulated the results obtained with the full dataset (mutation rates: 2.0 × 10^−3^ and 5.2 × 10^−3^ for rapid and normal progressors, respectively; Mann-Whitney test: *p* = 0.018), the situation was reversed for low-level editing, with rapid progressors showing a 3.2-fold increase in mutation rate compared to normal progressors (4.2 × 10^−4^ and 1.3 × 10^−4^, respectively; *p* = 0.043; Fig 4E). Similarly, in contrast to the total mutation rate, the low-level A3 editing rate correlated positively with set-point viral load (Spearman *ρ* = 0.606; *P* = 0.048; Fig 4F). Notice that the low-level A3 editing rate was still at least twice as high as the RT mutation rate. Our results thus support a dual role for A3 in disease progression. On one hand, A3 exert an antiviral effect by introducing a large number of mutations in the HIV-1 genome, which usually abolish infectivity. On the other hand, though, a less extensive edition does not ensure the loss of infectivity and may promote pathogenicity.

## Discussion

The rate of spontaneous mutation is a major determinant of viral diversity and evolution, plays a role in the success of vaccination strategies [43,44], determines the likelihood that live attenuated vaccines revert to virulence [45], and influences the risk of disease emergence at the epidemiological level [46,47]. In the last years, direct experimental evidence has established that the viral mutation rate is also a virulence factor. For instance, a poliovirus with a high-fidelity polymerase showed lower ability to evolve drug resistance and to escape antibody neutralization in cell culture and was significantly attenuated in mice as a result of its impaired ability to evade the immune response or to adapt to different microenvironments in vivo [48,49]. Similar results have been obtained with a high-fidelity variant of chikungunya virus [50] and, recently, with other fidelity variants of poliovirus [51] and enterovirus 71 [52], suggesting that RNA viruses have optimized their mutation rates for maximal adaptability and underscoring the importance of quantifying viral mutation rates in vivo. To date, this has been largely done under cell culture conditions for different RNA viruses, including HIV-1 and other retroviruses, influenza virus, measles virus, poliovirus, plant viruses, and bacteriophages and has indicated that RNA virus mutation rates range from 10^−6^ to 10^−4^ per base per cell [4]. In contrast, quantitation of these rates in vivo has been more problematic. The only available estimates for a human virus in vivo correspond to hepatitis C virus (HCV) and are consistent with the values obtained for other RNA viruses in cell culture [53,54].

Inference of the rate of spontaneous mutations in vivo is complicated by the unknown number of viral generations (i.e., infection cycles), the removal of a large number of deleterious mutations by selection, and genetic drift, among others. By focusing on a single cell infection cycle, the lethal mutation method bypasses most of these uncontrolled factors. The assumption that sequences carrying stop codons will not undergo subsequent infection cycles is substantiated by the essential or quasiessential nature of all HIV-1 genes in vivo. However, some stop codon mutants may be able to complete multiple infection cycles if they are genetically complemented by other genomes coinfecting the same cells, as has been shown for some specific stop codon mutations in other RNA viruses [55]. However, if this process was widespread in HIV-1, the abundance of stop codons in sequences from plasma would approach that of viral DNA sequences, but our results clearly show that this does not occur. Another potential bias may come from the fact that CD4 cells infected with stop codon-containing, defective proviruses may have a longer lifespan than those infected with nondefective viruses, thus becoming overrepresented in the PBMC population. However, HIV-1 genome integration typically requires that the host cell is activated, because otherwise nucleotide pools are too low to support efficient reverse transcription and nuclear transport of the preintegration complex [56], but activated lymphocytes have short lifespans even if they are not infected [57]. Additionally, it has been estimated that only 1/5 to 1/10 of the HIV-1 DNA is comprised of integrated proviral genomes and thus that most viral DNA is short-lived [58,59]. Therefore, usage of stop codons for inferring the HIV-1 mutation rate should not be a major source of bias. Supporting this view, application of this same method to HCV yielded estimates that are within the accepted range for RNA viruses [53,54], and our inference from plasma RNA sequences is also consistent with this range [4,6–9].

**Fig 5:**
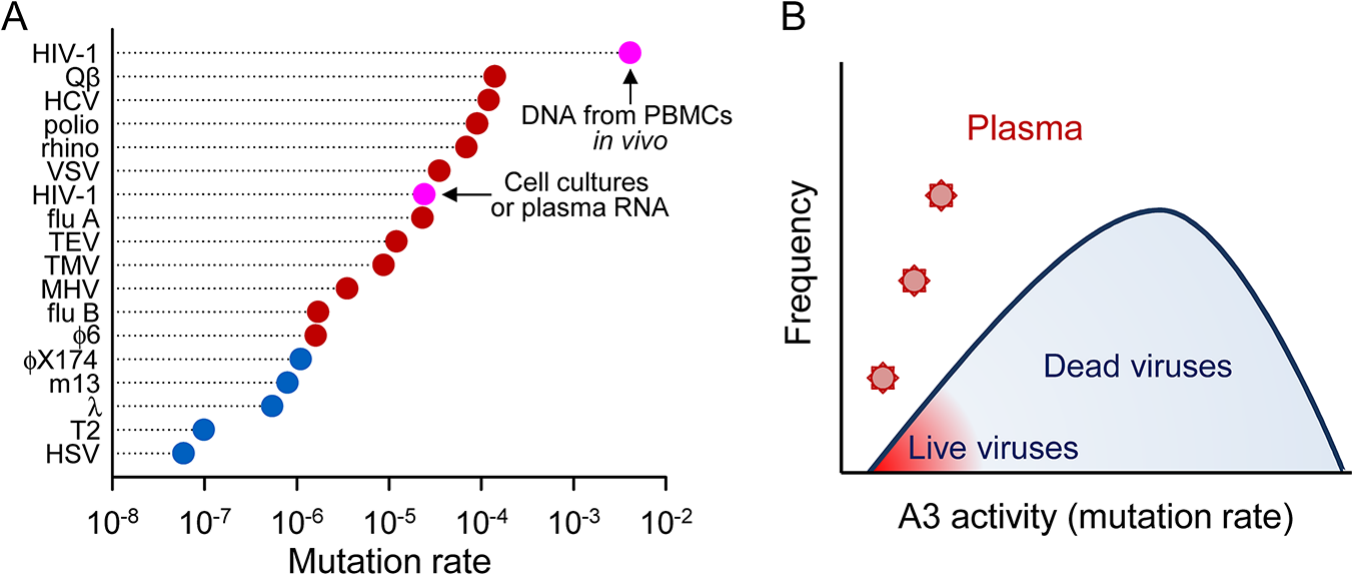
Comparison of viral mutation rates and effect of A3-mediated editing. **A.** Mutation rates per base per cell are shown for HIV-1, bacteriophage Qβ, HCV, poliovirus, human rhinovirus 14, vesicular stomatitis virus (VSV), influenza A virus, tobacco etch virus (TEV), tobacco mosaic virus (TMV), murine hepatitis virus (MHV), influenza B virus, bacteriohages ϕ6, ϕX174, m13, λ and T2, and herpes simplex virus 1 (HSV). RNA viruses are shown in red and DNA viruses in blue. Two HIV-1 data points are shown in pink, one obtained from cell culture studies [4] (which is similar to the estimate obtained here using plasma RNA), and the estimate obtained here from PBMC DNA. All other mutation rates were taken from a review [4] except for Qβ [63]. Numerical values can be retrieved from these references. Other reverse-transcribing viruses are believed to exhibit mutation rates similar to those of RNA viruses, but these are not shown because previous work did not address the potentially strong contribution of A3 in vivo. Notice that the mutation rate axis is in log-scale, such that the HIV-1 mutation rate is 37 times higher than the second highest rate, and also substantially higher than the rate obtained from plasma RNA or under cell culture conditions. **B.** According to our interpretation, this discrepancy occurs because most A3-edited sequences are lethally mutated and are thus unable to reach the plasma. Therefore, analysis of plasma RNA sequences would lead to a gross underestimation of the actual HIV-1 mutation rate.

In contrast, our results using DNA from PBMCs provide a much higher mutation rate for HIV-1, which clearly deviates from the accepted range of rates for RNA viruses (Fig 5A). This extremely high mutation rate is essentially driven by A3 editing, the relative contributions of A3G, A3D/F/H, and RT being approximately 100:10:1, respectively. Hypermutation should be largely lethal for the virus, particularly considering the low robustness exhibited by some HIV-1 proteins [60,61]. This is consistent with the observation that 88% of latently integrated proviruses are genetically defective [62]. However, low-edited viruses may be able to form infectious particles and reach the plasma (Fig 5B).

As we have shown, A3 enzymes still contribute approximately 50% of the spontaneous mutations in plasma. Furthermore, since these sequences were obtained by RT-PCR and given that RTs tend to exhibit considerably lower fidelity in vitro than in vivo [35,36], it is likely that the actual contribution of A3 to sequence diversity in plasma is greater than estimated here. Previous analyses of intrapatient viral diversity have suggested a role for A3 in promoting immune escape and drug resistance [17,18], and antiviral therapy failure was found to be more frequent among patients infected with defective *vif* alleles [64]. Our results showing that A3 introduces enormous numbers of mutations in HIV-1 but that the amount of A3 editing varies support such as dual role for A3 as an antiviral factor and a diversity-generating agent. The correlations found with disease progression markers underscore the importance of viral mutation rates for pathogenesis. However, noncausal relationships between viral mutation rates and disease progression may be envisaged. For instance, variants showing different cell tropism may be subject to varying editing rates depending on A3 expression levels in the host cell type. Since cell tropism and disease progression are associated [65,66], this might produce a correlation between the HIV-1 mutation rate and disease progression. Computational analysis of consensus V3-loop sequences suggested CCR-5 coreceptor usage in all patients except R5 and, therefore, we found no evidence that systematic differences in cell tropism among patients may drive the observed correlations.

In conclusion, we have inferred an extremely high mutation rate in HIV-1 patients, which is mainly caused by A3-driven editing of the viral genome. We argue that analysis of sequences from plasma grossly underestimates the HIV-1 mutation rate due to the abundance of lethal mutations that are incompatible with the release of viral particles. A3 proteins have also been shown to mutate hepatitis B virus [67] and nonreverse transcribing DNA viruses such as papillomaviruses [68] or herpesviruses [69]. In addition, the double-stranded RNA-specific adenosine deaminase (ADAR) can edit the genomes of many RNA viruses including measles virus [70], human parainfluenza virus [71], respiratory syncytial virus [72], lymphocytic choriomeningitis virus [73], and Rift Valley fever virus [74]. Future work may elucidate whether intracellular sequences also harbor higher-than-average mutation rates in these viruses, similar to A3-edited HIV-1 sequences. As shown for HIV-1, host-mediated hypermutation of viral genomes can be regarded as an antiviral mechanism, but a downside of this process is that it may contribute to viral genetic diversity and pathogenesis. In our study, we have focused only on normal and rapid progressors, but it remains unclear whether similar results will be observed in other disease progression categories such as long-term nonprogressors or elite controllers.

## Methods

### Patients

Ten samples from patients were kindly provided by the HIV BioBank integrated in the Spanish AIDS Research Network (RIS) [75], and one sample (P6) was provided by Hospital La Fe (Valencia, Spain). Samples were processed following standard procedures and frozen immediately upon reception. For sample P6, 10 mL of blood was provided by Hospital La Fe (Valencia, Spain), and DNA extraction from PBMC was performed following buffy coat purification. For all samples, approximately 10 million PBMCs were used for DNA extraction using QIAamp DNA Blood Mini Kit (Qiagen). All patients participating in the study gave their informed consent, and protocols were approved by institutional ethics committees. The clinical and epidemiological data provided for patients were included in the cohort of adults with HIV infection of the AIDS Research Network (CoRIS). The program was approved by the Institutional Review Boards of the participating hospitals and centers. The cohort of adults with HIV infection of the AIDS Research Network (CoRIS) is an open, multicenter cohort of patients newly diagnosed with HIV infection at the hospital or treatment center, over 13 years of age, and naïve to antiretroviral treatment. The information is subject to internal quality controls; once every two years, information on 10% of the cohort is audited by an externally contracted agency.

### Amplification of Viral DNA by Limiting Dilution PCR

Nearly complete HIV genomes were amplified in three overlapping PCR fragments (named as regions 1, 2, and 3) using different sets of primer described previously [76], and which are provided in S1 Table. High-fidelity limiting dilution PCR was performed using Phusion polymerase (Thermo Scientific) to amplify clonal sequences, setting the dilution of the sample such that the percentage of positive PCRs was about 10%. Primary PCRs were performed at 2 min at 98°C, 35 cycles of 5 s at 98°C, 30 s at 62°C, and 2 min at 72°C, and a final extension of 10 min at 72°C. Secondary amplification was done by nested PCR under the same conditions but with an annealing temperature of 65°C. Positive reactions were picked from the 96-well plates and visually checked in agarose gels for equal concentration. For each patient, fifty clonal PCR products were obtained for each region. PCR products were pooled as indicated, purified using High Pure PCR Product Purification Kit (Roche), and sequenced by Illumina HiSeq2000 using paired-end libraries (Genoscreen, France).

### NGS Sequence Mapping and Mutation Calling

Fastq files were cleaned and trimmed using FASTX toolkit version 0.0.14 and dereplicated using Prinseq-lite version 0.20.3 [77]. To obtain the consensus for each patient, 50,000 paired reads were subsampled from each library, pooled, and mapped using Bowtie 2 version 2.2.4 [78] to reference libraries generated from overlapping half-genomes alignments (position 1–5,000 and 4,000–end) of 6 subtype B reference sequences (HXB2, pNL4.3, K03455, AY423387, AY173951, AY331295). Reads mapping to each region were then split using a custom script and the consensus sequence obtained using VICUNA [79] with default settings. Aligning reads to each half genome region was necessary to properly assemble the 5' and 3' UTR regions. Contigs from both regions were then merged using the contigMerger.pl script of V-FAT (Broad institute) to generate the overall consensus for each patient, which were deposited in GenBank (accession numbers KT200348-KT200358). Using these references, reads were mapped using MOSAIK-2.2.3 aligner [80]. V-profiler [81] was then used to call mutations at each codon position, excluding mutations occurring only at the last 10 bases of reads. The codon details output file from V-phaser 2 was then parsed using a custom R script to keep only codons that have <30% low quality reads, and a mutation frequency >6%. Positions with stop codons were then extracted and their occurrence in each mix of five clones estimated using the formula 6%–30% = 1 genome, 31%–50% = 2 genomes, 51%–70% = 3 genomes, and 71%–90% = 4 genomes. In addition, a consensus sequence for each mix (mix consensus) was obtained from the nucleotide frequency output file of V-phaser 2 and an overall consensus for each patient derived from these.

### Sanger Sequencing of *nef* Clones

Positive primary PCRs of fragment 3 from libraries showing at least two stop codons per pool as determined by NGS were reamplified, column-purified, and subjected to Sanger sequencing using the reverse PCR primer. Chromatograms were analyzed using the Staden package (version 2.0.0b10). Sequences were converted to Fasta using Egglib software (version 2.1.7), and the number and nature of stop codons determined by visual inspection in Aliview (version 1.17.1). Sequences were deposited in GenBank (accession numbers KT205403–KT205555).

### Sequences from Plasma RNA

For *env* gene, two available datasets of full length, codon alignments were downloaded, one of subtype B (http://www.hiv.lanl.gov/content/sequence/HIV/USER_ALIGNMENTS/keele.html) and another of subtype C (http://www.hiv.lanl.gov/content/sequence/HIV/SI_alignments/set5.html). These were separated into individual alignments for each patient including the precalculated consensus using a custom script. For *gag*, sequences were downloaded from the HIV database that encompassed the entire gene, included at least four sequences per patient, and were derived by single genome amplification, cloning, or limiting dilution. The *gag* region was then obtained using the GeneCutter tool available from this database, and sequences were codon-aligned using HIV Align with HMM-align option. Sequences from individual patients were then separated, and a consensus calculated using Biostrings R package. For each patient in the three datasets, sequences with frameshifts were removed.

### Calculation of A3G, A3D/F/H, and RT Mutation Rates

NSMTs are codons that can mutate to a stop codon via a single nucleotide substitution [53,54]. The presence of these codons was identified in each reference sequence and their abundance calculated by multiplying by the number of sequences for each patient. Based on sequence preference of A3G (GG → AG) or A3D/F/H (GA → AA), NSMTs were assigned as potential targets for A3G, A3D/F/H, or RT (see Table 1), with all mutations which do not occur at A3G or A3D/F/H sequence motifs being assigned to the RT category. Subsequently, all stop codons were identified and assigned as an A3G, A3D/F/H, or RT mutation (e.g., TAG codon in TGG is A3G; CAG to TAG is RT). Mutation rates for A3 enzymes were calculated as the number of A3 mutations per A3 NSMT. Mutation rates for RT were calculated similarly but were multiplied by three to account for the fact that only one out of three bases at each NSMT produces an observable stop codon. In all cases, the last 5% of each gene was not considered in order to avoid inclusion of stop codons with reduced effect on protein function. Finally, for three patients, a single PCR fragment had a mutation rate that was <3% of the other 2 PCR fragments. These are likely due to selective amplification of nonhypermutated genomes due to primer mismatches and were therefore not included in the analysis.

### A3H Haplotype Determination

PCR was performed on patient DNA to amplify exon 3 of A3H using primers 5-CATGGGACTGGACGAAACGCA (A3H105F) and 5-TGGGATCCACACAGAAGCCGCA (A3H105R), Phusion high-fidelity polymerase, and 35 cycles. The resulting PCR was directly sequenced to ascertain the presence of a glycine or arginine at amino acid position 105 (reference SNP 139297). For patients homozygous (P6, R7, and R8) or heterozygous (R15) for an arginine at position 105, indicating a potentially active haplotype, PCR was performed to amplify A3H exon 2 using primers 5-GTGGCTTGAGCCTGGGGTGA (A3H15F) and 5-CAGAGAGCCCGTGTGGCACC (A3H15R). The PCR product was then cloned, and 5 clones per patient were analyzed for the presence of a deletion at amino acid position 15 (reference SNP 79323350). With the exception of R15, all patients were homozygous for a deletion at position 15, indicating an unstable A3H genotype. R15 was homozygous for the presence of an asparagine at position 15 and hence carried one stable and one unstable allele.

### Coreceptor Usage Prediction

V3 loop sequences from the consensus sequence from each patient were analyzed using WebPSSM tool (http://indra.mullins.microbiol.washington.edu/webpssm/), and only R5 was predicted to use CXCR4.

## Supporting Information

S1 Data Average mutation rates and clinical information for each patient. Total, A3G, A3D/F/H, low-level A3, and high-level A3 mutation rates are provided. Mutation rates inferred from plasma RNA are also provided. Sequences from PBMC DNA were obtained by Illumina sequencing in this study. Consensus sequences for each patient are available from Genbank accessions KT200348–KT200358. Sequences from plasma RNA are publicly available at http://www.hiv.lanl.gov/content/sequence/HIV/USER_ALIGNMENTS/keele.html and http://www.hiv.lanl.gov/content/sequence/HIV/SI_alignments/set5.html.
(XLSX)

S2 Data Detailed clinical data. The infection time, viral load, and CD4 count are provided for each patient sample.
(XLSX)

S3 Data List and location of stop codons in each patient. For each patient (columns), genes, NSMT-containing codons, and the observed number of stop codons are shown.
(XLSX)

S4 Data List of stop codons found in each sequencing library. For each patient and sequencing library, the total, A3G, A3D/F/H, and RT stops are indicated, and libraries are classified depending on the A3 mutation level (high/low).
(XLSX)

S5 Data Stop codons in individual *nef* sequences. Each library containing at least two stops was analyzed by sequencing the *nef* gene of each of the five clones (i.e., limiting-dilution PCRs) constituting the library by the Sanger method. The patient, library, and number of stop codons found in each clone are shown. The observed number of clones with zero stops is compared with the number expected under a Poisson model, and a *p*-value is provided for each library. Sequences are available from Genbank accessions KT205403–KT205555.
(XLSX)

S1 Fig CD4 counts of the 11 patients included in this study. The per-year CD4 count decay rate was obtained by linear regression (dashed lines). The numerical values shown in this Figure are provided in the S2 Data file.
(TIF)

S2 Fig Viral load determinations for the 11 patients included in this study. The set-point viral load was obtained by averaging the log load values obtained at least one year postinfection (dashed lines). The numerical values shown in this Figure are provided in the S2 Data file.
(TIF)

S3 Fig Distribution of stop codons and NSMTs across HIV-1 genes. Total, A3G, A3D/F/H, and RT stop codons, NSMTs within a sliding window of 50 codons (black skyline), and the average for each gene (red dashed line) are shown. Numerical values can be obtained from the S3 Data file.
(TIF)

S4 Fig Distribution of the number of stop codons per library in *nef*. Each column corresponds to a sequencing library showing at least two total *nef* stop-codons from the indicated patient. Each stacked bar shows the number of stop codons found in each clone of that library (clones with no stops are not represented). Asterisks indicate libraries in which mutations were significantly clustered in a subset of clones. Numerical values are provided in the S5 Data file.
(TIF)

S5 Fig Distribution of mutation rates across HIV-1 genes in rapid and normal progressors. The mutation rate within a sliding window of 50 codons (skylines) and the average for each gene (dashed lines) are shown for rapid (red) and normal (blue) progressors. Numerical values can be obtained from the S3 Data file.
(TIF)

S1 Table List of primers used for limiting-dilution PCR. (XLSX)

## Abbreviations

A3: apolipoprotein B mRNA editing enzyme, catalytic polypeptide-like 3
ADAR: double-stranded RNA-specific adenosine deaminase
HCV: hepatitis C virus
HSV: herpes simplex virus 1
MHV: murine hepatitis virus
NSMT: nonsense mutational targets
PBMC: peripheral blood mononuclear cells
RT: reverse transcriptase
TEV: tobacco etch virus
TMV: tobacco mosaic virus
Vif: viral infectivity factor
VSV: vesicular stomatitis virus
