# Supplementary figures and images for: Extremely High Mutation Rate of HIV-1 In Vivo
Source: PLoS Biol. 2015 Sep 16;13(9):e1002251. doi: 10.1371/journal.pbio.1002251 (PMC4574155; doi:10.1371/journal.pbio.1002251)

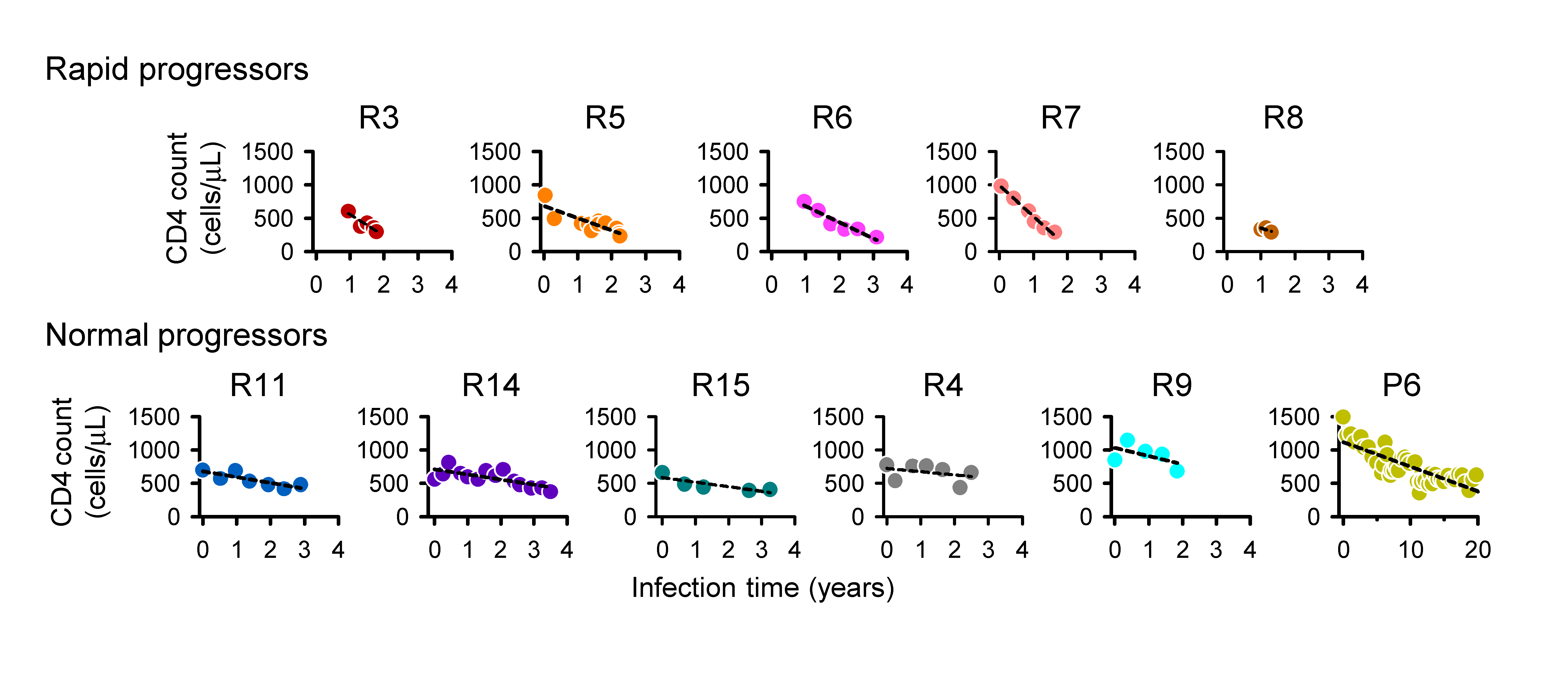

Supplement: S1 Fig — The per-year CD4 count decay rate was obtained by linear regression (dashed lines). The numerical values shown in this Figure are provided in the S2 Data file. (TIF) [file pbio.1002251.s006.tif]

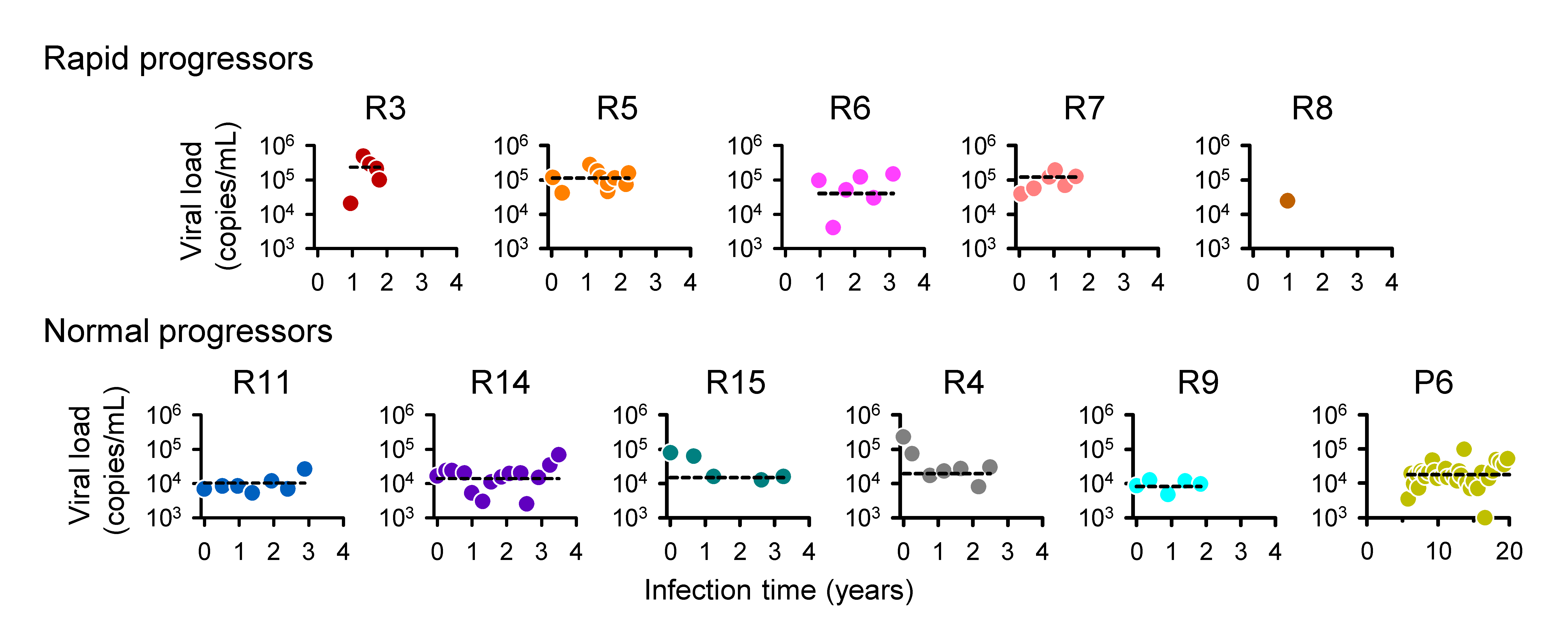

Supplement: S2 Fig — The set-point viral load was obtained by averaging the log load values obtained at least one year postinfection (dashed lines). The numerical values shown in this Figure are provided in the S2 Data file. (TIF) [file pbio.1002251.s007.tif]

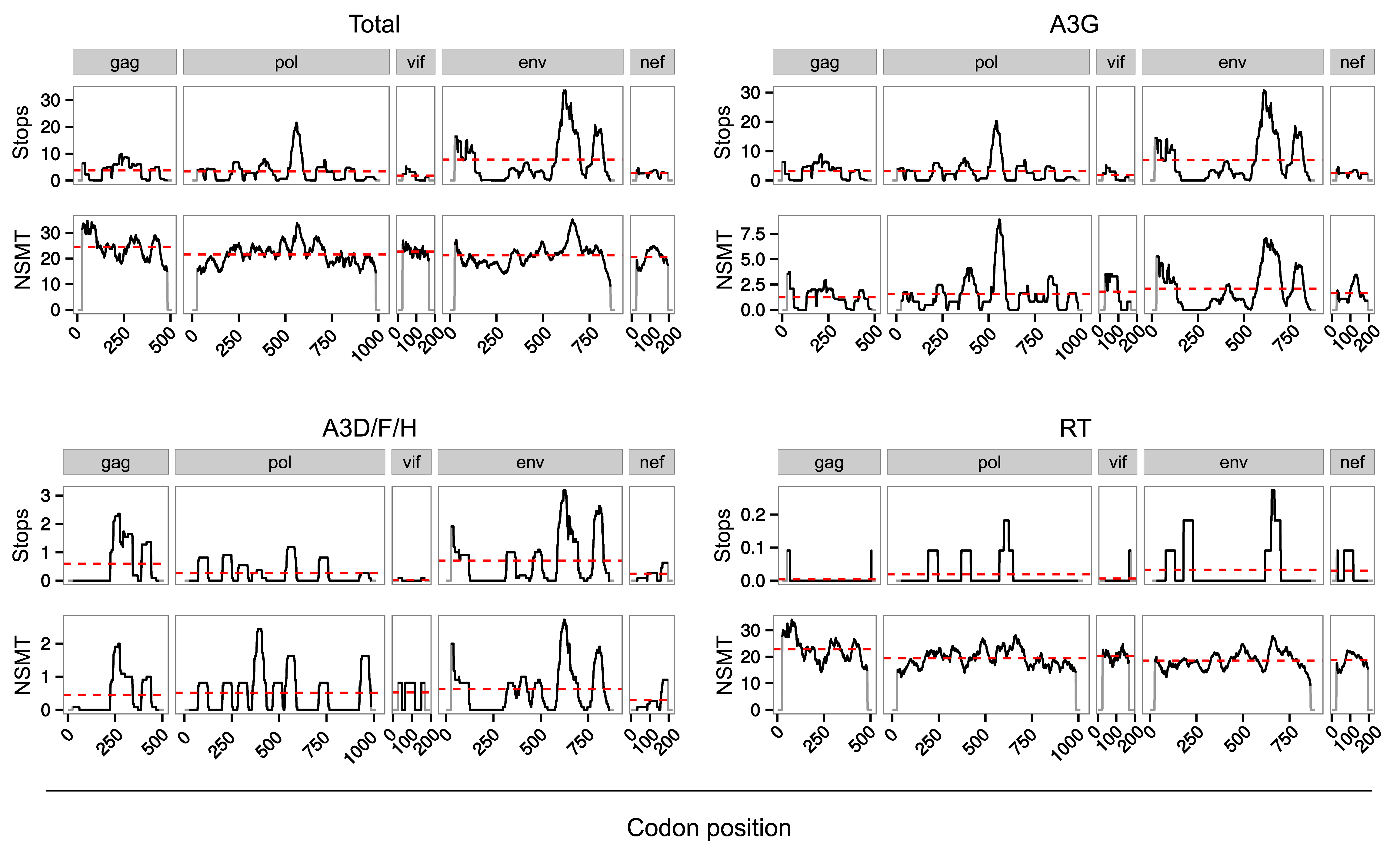

Supplement: S3 Fig — Total, A3G, A3D/F/H, and RT stop codons, NSMTs within a sliding window of 50 codons (black skyline), and the average for each gene (red dashed line) are shown. Numerical values can be obtained from the S3 Data file. (TIF) [file pbio.1002251.s008.tif]

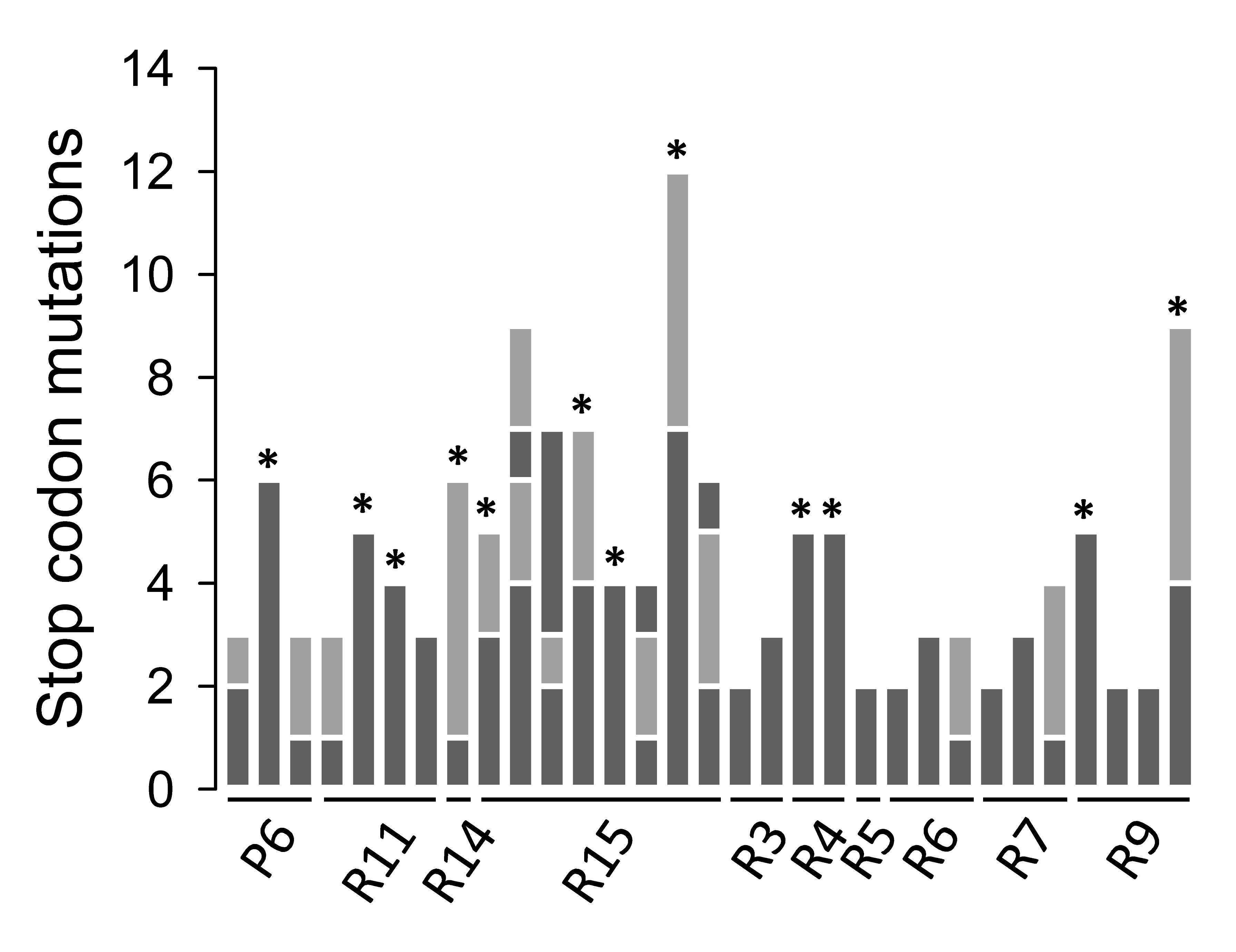

Supplement: S4 Fig — Each column corresponds to a sequencing library showing at least two total nef stop-codons from the indicated patient. Each stacked bar shows the number of stop codons found in each clone of that library (clones with no stops are not represented). Asterisks indicate libraries in which mutations were significantly clustered in a subset of clones. Numerical values are provided in the S5 Data file. (TIF) [file pbio.1002251.s009.tif]

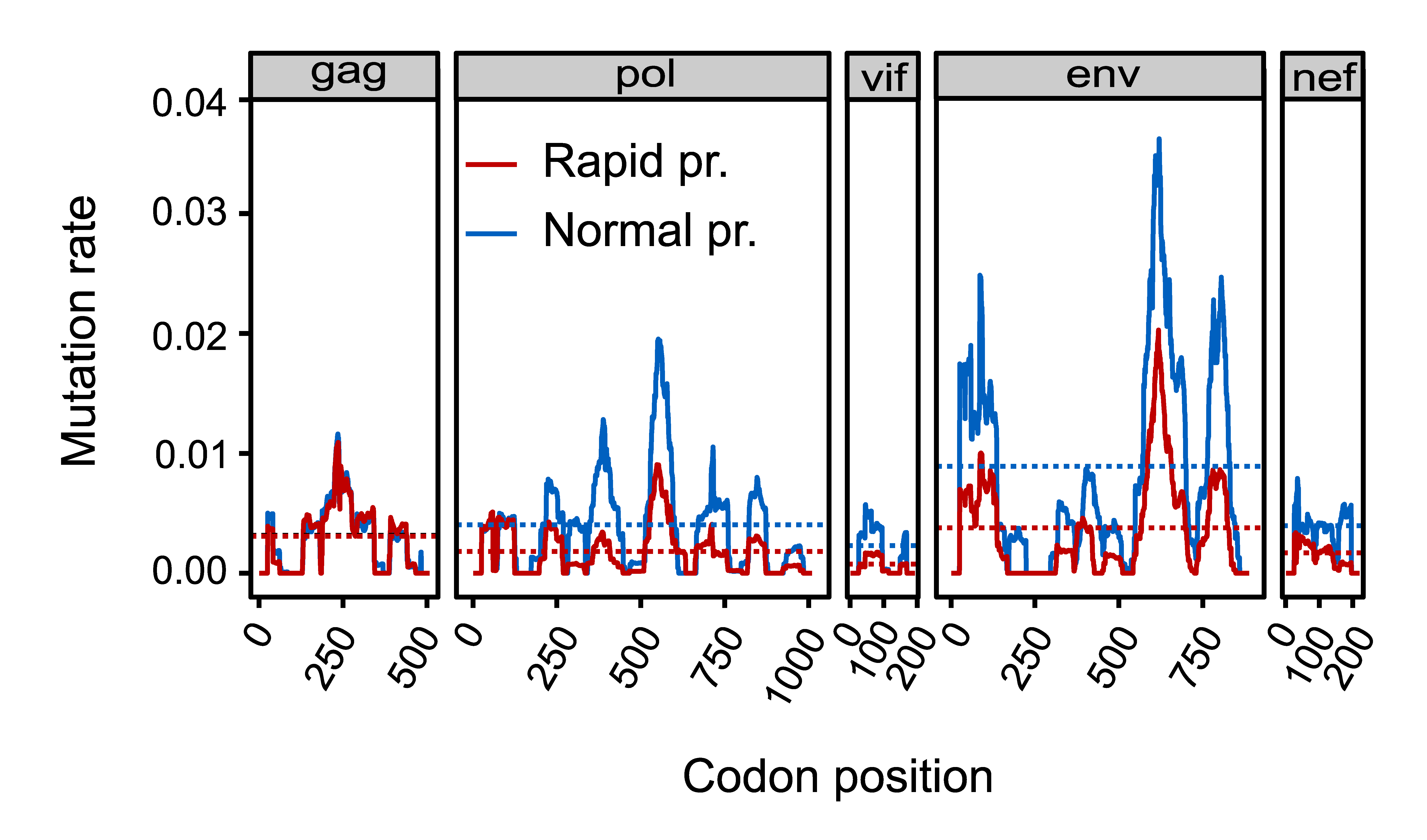

Supplement: S5 Fig — The mutation rate within a sliding window of 50 codons (skylines) and the average for each gene (dashed lines) are shown for rapid (red) and normal (blue) progressors. Numerical values can be obtained from the S3 Data file. (TIF) [file pbio.1002251.s010.tif]
